# Users’ experience with health-related content on YouTube: an exploratory study

**DOI:** 10.1186/s12889-023-17585-5

**Published:** 2024-01-03

**Authors:** Fatma Mohamed, Abdulhadi Shoufan

**Affiliations:** Center for Secure Cyber-Physical Systems, Khalifa University, Abu Dhabi, United Arab Emirates

**Keywords:** YouTube, Health-related videos, Medical videos, Content quality, Decision-making

## Abstract

**Background:**

This study focuses on health-related content (HRC) on YouTube and addresses the issue of misinformation on this platform. While previous research centered on content evaluations by experts, this study takes a user-centered approach and aims to explore users’ experiences with and perceptions of HRC videos and to establish links between these perceptions and some socio-demographic characteristics including age, gender, profession, and educational level.

**Methods:**

A quantitative research design was used in the study. 3,000 YouTube users responded to a 35-item anonymous questionnaire to collect information about the content they watch toward decision-making, their perceptions of the usefulness and bias of this content, what they identify as quality indicators for HRC, and what they recommend to improve the quality of such content on YouTube. The data were analyzed using descriptive statistics, frequency, and correlation analyses.

**Results:**

The results reveal that 87.6 percent (n=2630) of the participants watch HRC on YouTube, and 84.7 percent (n=2542) make decisions based on what they watch. Exercise and bodybuilding videos are the most popular, with over half of the participants watching them. 40 percent of the users watch YouTube videos to decide whether to consult a doctor or adopt specific health-related practices. In contrast to evaluations by experts in previous studies, most respondents perceive HRC videos on YouTube as useful and do not find connections between video quality and surface features like the number of views and likes. Weak or no correlations were observed between the perceived usefulness of HRC videos and age, gender, profession, or educational level. Participants’ recommendations for enhancing HRC quality align with previous research findings.

**Conclusions:**

Users turn to YouTube not only for health information but also as a decision-making tool. Combined with their generally positive attitudes towards content quality on this platform, this can have significant consequences for their health. Follow-up studies are needed to get more insights into decision-making behaviors and how users assess their decisions in retrospect.

**Supplementary Information:**

The online version contains supplementary material available at 10.1186/s12889-023-17585-5.

## Background

YouTube has emerged as a significant source of health-related information [1, 2]. According to the Health Information National Trends Survey (HINTS) conducted by the National Cancer Institute, the percentage of U.S. adults who watched health-related YouTube videos in the last 12 months has increased from 39.7% in 2020 (HINTS 5 CYCLE 4) to 58.9% in 2022 (HINTS 6) [3, 4]. This surge in viewership reflects a growing trend of individuals actively engaging with health-related content (HRC) on the platform. People are not only consuming information but also utilizing YouTube as a platform to seek support from others who share similar health conditions, find answers to their health-related questions, and even gather information prior to planned medical procedures [5, 6]. In a study by Kimberly Haslam et al., various motivations were identified for seeking health information on YouTube, including self-diagnosis support, enhancing knowledge about medical conditions and procedures, alleviating anxiety, exploring treatment options, understanding the purpose and potential side effects of specific medications, finding social support, and accessing information on health programs and services [7]. These findings emphasize the diverse needs and intentions of users when engaging with HRC on YouTube.

The impact of YouTube on users’ health-related behaviors and decisions is substantial. Numerous studies in the literature have emphasized the effectiveness of online health-related content in assisting users with their health decisions. One notable study conducted by Maria Bujnowska-Fedak and Paulina Wegierek highlighted the profound influence of internet-derived health and disease information on patients’ decisions and behaviors [8]. More than 50% of the surveyed users reported that online health-related content influenced their decisions regarding diet and physical activity. Additionally, 45% of users scheduled appointments with healthcare professionals, and 40% sought answers to medical questions based on the information obtained online. Another study by Juhan Lee et al. demonstrated that watching health-related videos on YouTube led to a significant increase of 30% in physical activity levels among U.S. adults [4]. According to a study by Kimberly Haslam et al. [7], reliable videos on YouTube have demonstrated their potential to effectively assist in public health decision-making. However, a significant challenge lies in ensuring the accessibility of reliable content for users seeking accurate health information. When users search for HRC on YouTube, they are frequently presented with a diverse list of results, where the quality and reliability of information can vary significantly. This potential discrepancy between popularity and content quality highlights the challenge of guaranteeing the availability and accessibility of reliable, high-quality health-related videos on the platform.

YouTube hosts a diverse array of health-related videos, encompassing both high-quality and low-quality content for public viewing [9, 10]. Consequently, users are left to navigate and discern the quality of the information they obtain from the platform [11]. This aspect becomes particularly crucial as it can significantly impact public health, given the popularity of health-related videos on the platform [12, 13].

In 2022, YouTube introduced the “YouTube Health” initiative that aims to help individuals find trustworthy sources of public health information. These videos are created by channels that adhere to principles established by the National Academy of Medicine (NAM) for the U.S., Council of Medical Specialty Societies (CMSS), reviewed by the American Public Health Association (APHA), and globalized guidelines from The World Health Organization (WHO) [14–16]. Through this initiative, YouTube has implemented several features to identify credible health-related content. When users watch a health-related video, an information panel is displayed, providing details about the source’s credibility. Furthermore, when users search for health-related topics, YouTube presents a reliable shelf of health content. This shelf includes a selection of credible videos based on the established source credibility principles. Additionally, when users search for specific health-related topics, a health information panel appears, offering information from authoritative resources such as the WHO and other reputable institutions, covering aspects like symptoms, prevention, and treatment of various diseases [17].

Numerous research studies investigated the problem of misinformation in HRC on YouTube [18–21]. However, the majority of these studies have primarily focused on evaluating the content quality of HRC videos from an expert standpoint using numerous quality evaluation standards such as the Journal of American Medical Association (JAMA) and DISCERN. What remains uninvestigated in the research is the users’ standpoint: How do users perceive HRC on YouTube and to what extent does it affect their health-related decisions? Understanding the users’ perceptions will help us estimate the potential impact of HRC videos on their health, given that low-quality content exists on the platform and the users have different levels of professional knowledge to judge the quality. This investigation will help us identify solutions and propose effective strategies to enhance the reliability and credibility of health-related content on the platform.

This study adopts a user-centered approach, aiming at providing a comprehensive understanding of the user experience with HRC videos on YouTube and comparing it with existing research findings. It provides the reader with an overview of the percentage of users seeking HRC videos on YouTube and making health-related decisions based on what they watch. This study also aims to provide valuable insights into how users perceive the overall usefulness and bias of HRC videos on YouTube. It also analyzes the correlation between the socio-demographic characteristics of the participants and the perceived quality of HRC on YouTube. Additionally, the study establishes connections between content quality and video surface features, such as video rank, channel popularity, and engagement metrics (views, likes, and comments), thereby providing a comprehensive analysis of the factors influencing users’ perceptions of content quality. Lastly, it explores users’ perspectives and level of agreement with research recommendations to enhance the content quality of HRC videos on YouTube. Specifically, the following research questions are addressed:

- **RQ1:** How many users watch HRC on YouTube, how frequently, and what do they watch exactly?
- **RQ2:** How many users make decisions based on HRC on YouTube, how frequently, and in what fields?
- **RQ3:** How do users perceive the quality of HRC on YouTube?
- **RQ4:** How do users link video surface features to content quality?
- **RQ5:** How does age, gender, profession or educational level affect the perceived usefulness of HRC on YouTube?
- **RQ6:** What do users recommend to mitigate the quality issue of HRC on YouTube?

Overall, this study offers a holistic perspective on the users’ experience with HRC videos on YouTube, shedding light on important aspects of online health information seeking, decision-making, and content quality assessment. The findings derived from this research will offer valuable insights for researchers, medical professionals, and content creators to develop effective strategies to enhance the reliability and credibility of HRC on YouTube and improve the overall user experience and health information accessibility on YouTube.

The rest of the paper is organized as follows. Methods section describes the methodology we followed in this study. Results section summarizes the research findings. In Discussion section, we discuss the results and compare them with previous research results, present a framework for future research, and describe the limitations of the study. Conclusion section concludes the paper.

## Methods

### Questionnaire design and development

The study utilizes a questionnaire-based quantitative research design. The survey consisted of 35 questions, but not all questions appeared to all participants. Some questions were displayed conditionally based on the participant answers to previous questions. The survey is divided into six parts to gather data about the demographic distribution of the participants, the type of HRC they watch, their decision-making, the overall perceived usefulness and bias of HRC videos on YouTube, the perceived indicators of content quality, and their recommendations to mitigate the issue of HRC quality on YouTube. Three question types were used including multiple-choice questions, multiple-answer questions, and rating questions on a 5-point Likert scale. The questions were presented to the participants once per page to reduce the cognitive load.

The questionnaire questions were derived based on a comprehensive literature review [22] that thoroughly examined 202 studies, focusing on the content quality of healthcare information in YouTube videos. The initial draft of the questionnaire was crafted by the authors of this study, and it then underwent multiple iterations. Two experts in the field critically reviewed and refined the questionnaire in multiple rounds to ensure the questions’ precision and clarity. Additionally, the survey was tested by a group of 15 selected individuals who provided useful feedback and comments that we incorporated into the final version of the questionnaire. The questionnaire was reviewed and approved by the Research Ethics Committee of our university.

#### Part 1: Demographic Information

Demographic information such as age, gender, nationality, and place of residency is available through the participant recruitment platform we used, as detailed in Participants and platform section. We linked this information to the participants’ responses through their ID that they provided as a response to the first survey question (Q1 in Appendix A [see Additional file 1]). In addition, we asked the participants about their highest educational degree and inquired whether their field of study or profession is related to health or medicine (Q2 and Q3 in Appendix A [see Additional file 1]).

#### Part 2: HRC Watching Behaviour

In this part, we first asked the participants whether and how frequently they use YouTube to watch HRC (Q4 in Appendix A [see Additional file 1]). The participants who indicated that they view such content were asked to select from a list all categories relevant to them (Q5 in Appendix A [see Additional file 1]). This list of health categories was derived based on the systematic review in [22]. On the other hand, participants who indicated that they don’t watch HRC on YouTube were asked to provide the reason for this (Q6 in Appendix A [see Additional file 1]).

#### Part 3: Decision-Making Behaviour

In this part, we first asked the participants whether and how frequently they make health-related decisions based on YouTube videos (Q7 in Appendix A [see Additional file 1]). Participants who confirmed that HRC on YouTube affects their decisions were asked to choose from a list of all types of decisions they make (Q8 in Appendix A [see Additional file 1]). The options on this list encompass general decisions, such as consulting a doctor, taking medicine, or accepting a treatment procedure, irrespective of the medical field. This list of decisions was developed based on the advice of two experts in this area.

#### Part 4: Perceived Usefulness and Bias

Users who indicated that they decide based on HRC on YouTube were asked to evaluate the usefulness of the YouTube videos in making these decisions (Q9-Q22 in Appendix A [see Additional file 1]). Furthermore, all users who watch HRC, as well as those who indicate that they don’t view such content due to quality concerns, were prompted to assess the general usefulness and bias of YouTube videos (Q23 and Q24 in Appendix A [see Additional file 1]). Usefulness and bias are frequently used to evaluate medical content on YouTube according to [22].

#### Part 5: Perception of Quality Indicators

The literature is rich in studies that try to establish links between content quality and some metrics including the video rank in the search list, the popularity of the channel [23], and the number of views, likes, and comments [22, 24, 25]. In this part of the survey, we asked the participants to rate how such factors affect the HRC on YouTube (Q25 and Q30 in Appendix A [see Additional file 1]).

#### Part 6: Recommendations

In their system review, Osman et al. presented a list of recommendations for mitigating the quality issue of HRC on YouTube [22]. In the final part of the survey, the participants were asked to rate the relevance of each of these recommendations (Q31 and Q35 in Appendix A [see Additional file 1]).

### Participants and platform

We recruited 3000 participants through a crowdsourcing platform called Prolific Academic (ProA) (https://prolific.com). ProA has been widely used for collecting research data reliably [26–28]. The platform allows researchers to set various criteria for participant selection. In our case, we specified that the participants must be regular users of YouTube and fluent in English. According to ProA, all registered members are at least 18 years old. The recruitment occurred on March 29, 2023, and the target number of 3000 participants was reached within 1 hour and 50 minutes. We provided the users with a brief description of the study and a consent form before being directed to the questionnaire hosted on Typeform (https://typeform.com/). The participants were informed that the survey was anonymous and their identities will be kept confidential. A monetary reward of approximately $\pounds$1.2 was paid to each participant.

### Data analysis

**Fig. 1:**
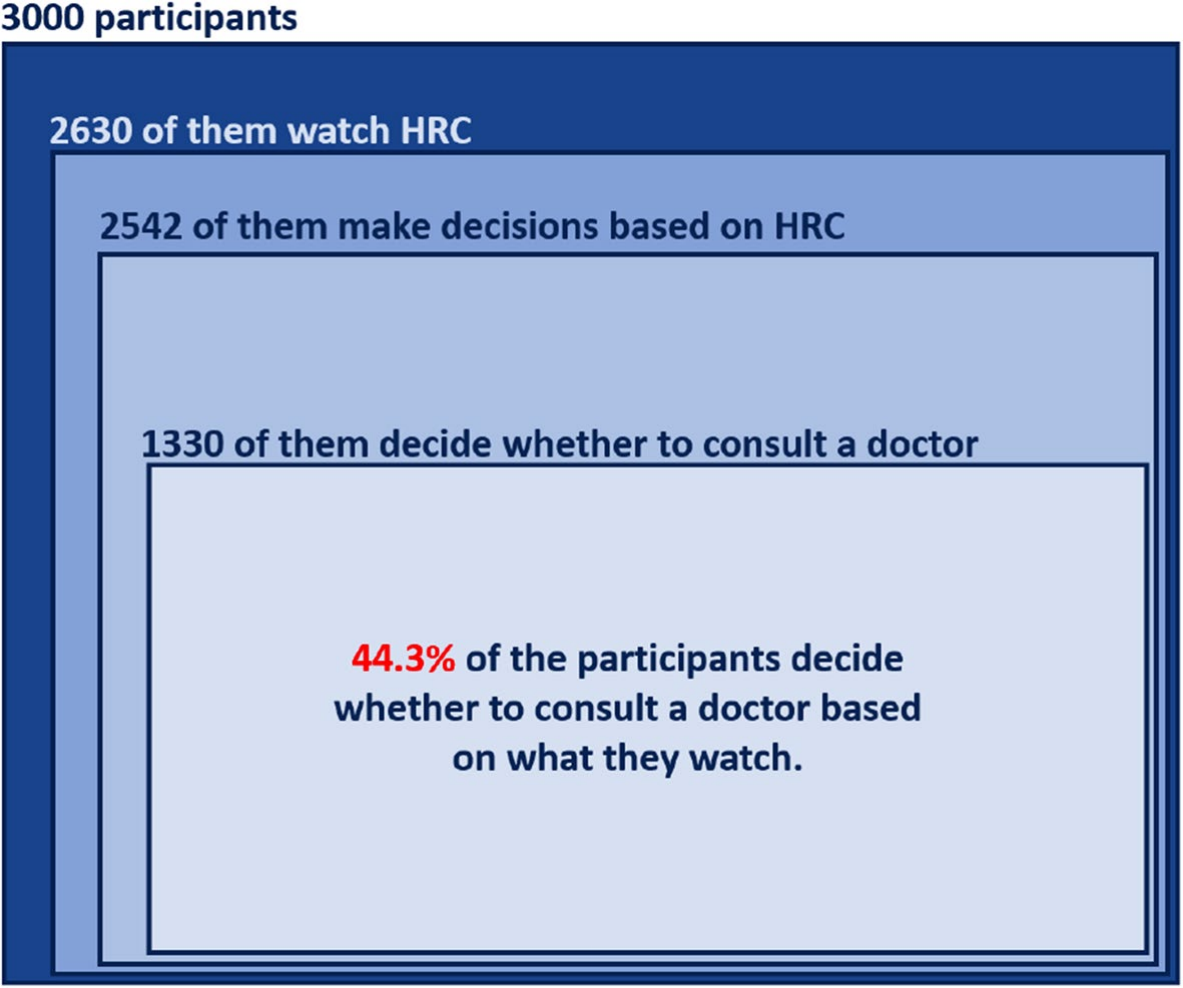
An example illustrating how the relative frequencies were calculated

To analyze the data, we used simple descriptive statistics, frequency analysis using bar charts, and correlation analysis to identify relationships. It is important to highlight that the relative frequencies were calculated with reference to the total number of participants, i.e., 3000. Figure 1 illustrates this aspect by an example. Here, 1330 of the participants reported that they decide whether to consult a doctor based on HRC they watch on YouTube. This figure makes $1330/3000 \times 100 = 44.3\%$ of all participants, $1330/2630 \times 100 = 50.6\%$ of those participants who watch HRC, and $1330/2542 \times 100 = 52.3\%$ of those participants who make decisions based on HRC. For consistent reporting, we opted to relate all the numbers to the total number of participants.

## Results

The survey was completed by 3000 participants, with 1533 (51.3%) females and an average age of 31.5 years, living in 33 countries on five continents (except Antarctica). 45.9% of the participants (n=1379) held a bachelor’s degree and 21.2% (n=636) a postgraduate degree. The remaining had a high school degree and a few of them had just completed elementary school. 28.6% (n=857) of the respondents worked in professions closely or partially related to health or medicine.

### RQ1-How many users watch HRC on YouTube, how frequently, and what do they watch exactly?

**Fig. 2:**
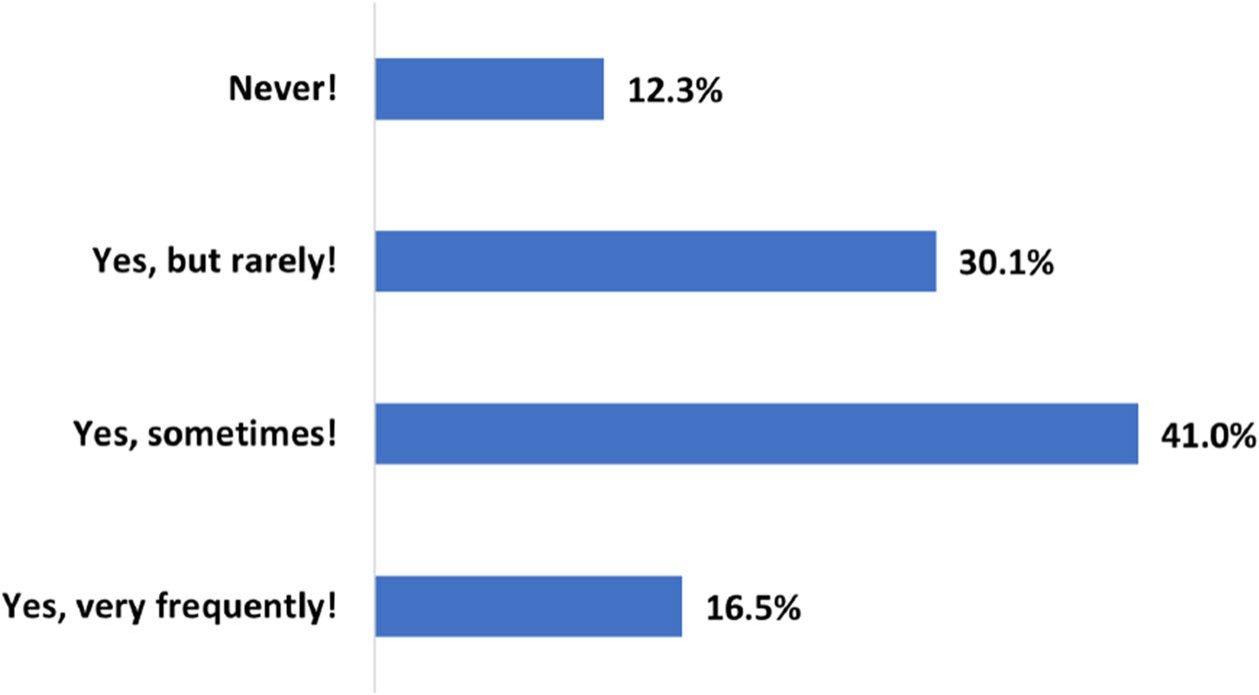
How frequent do users watch HRC videos on YouTube

Initially, the respondents were asked whether and how frequently they seek health information on YouTube. According to Fig. 2, 87.6% (n=2630) of them indicated that they watch HRC on this platform, and 16.5% (n=496) even do this very frequently. Only 12.3% (n=370) do not view medical or health videos on YouTube.

**Fig. 3:**
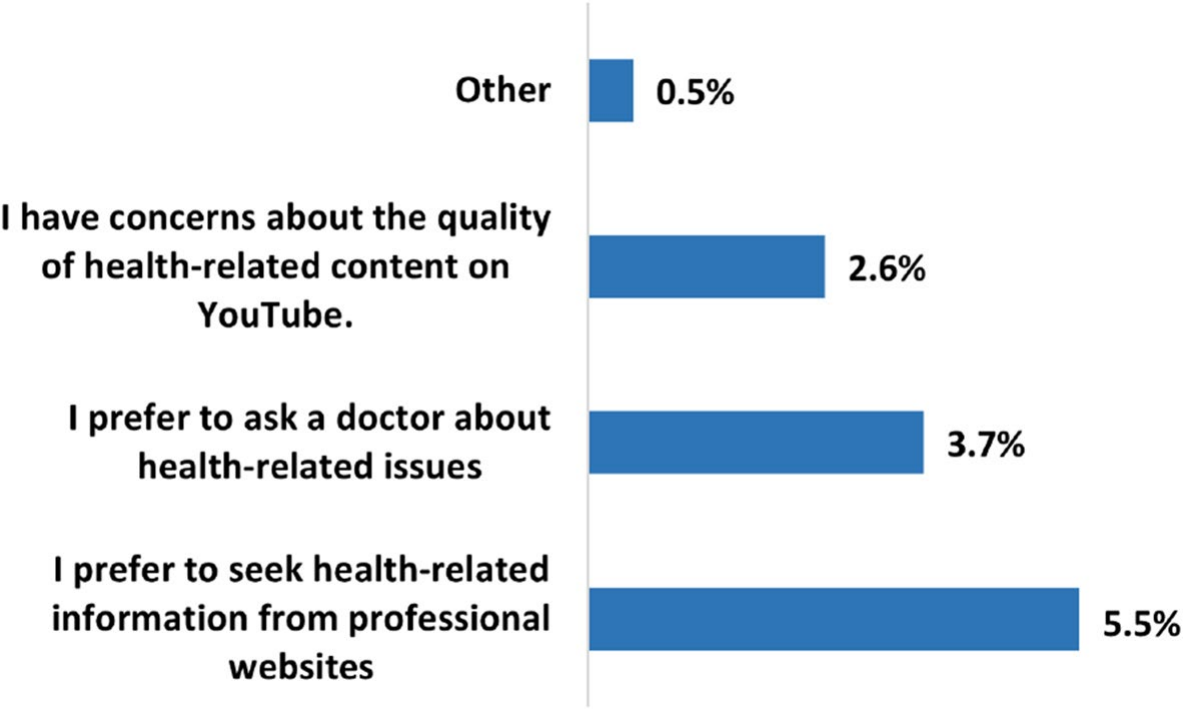
Why do some users refrain from using YT to seek health-related information?

Figure 3 summarizes the reasons why some users refrain from watching HRC on YouTube. Accordingly, 5.5% (n=164) expressed that they prefer to seek such information on professional websites, 3.7% (n=112) prefer to consult a doctor, and 2.6% (n=79) have concerns about the quality of HRC on YouTube.

**Fig. 4:**
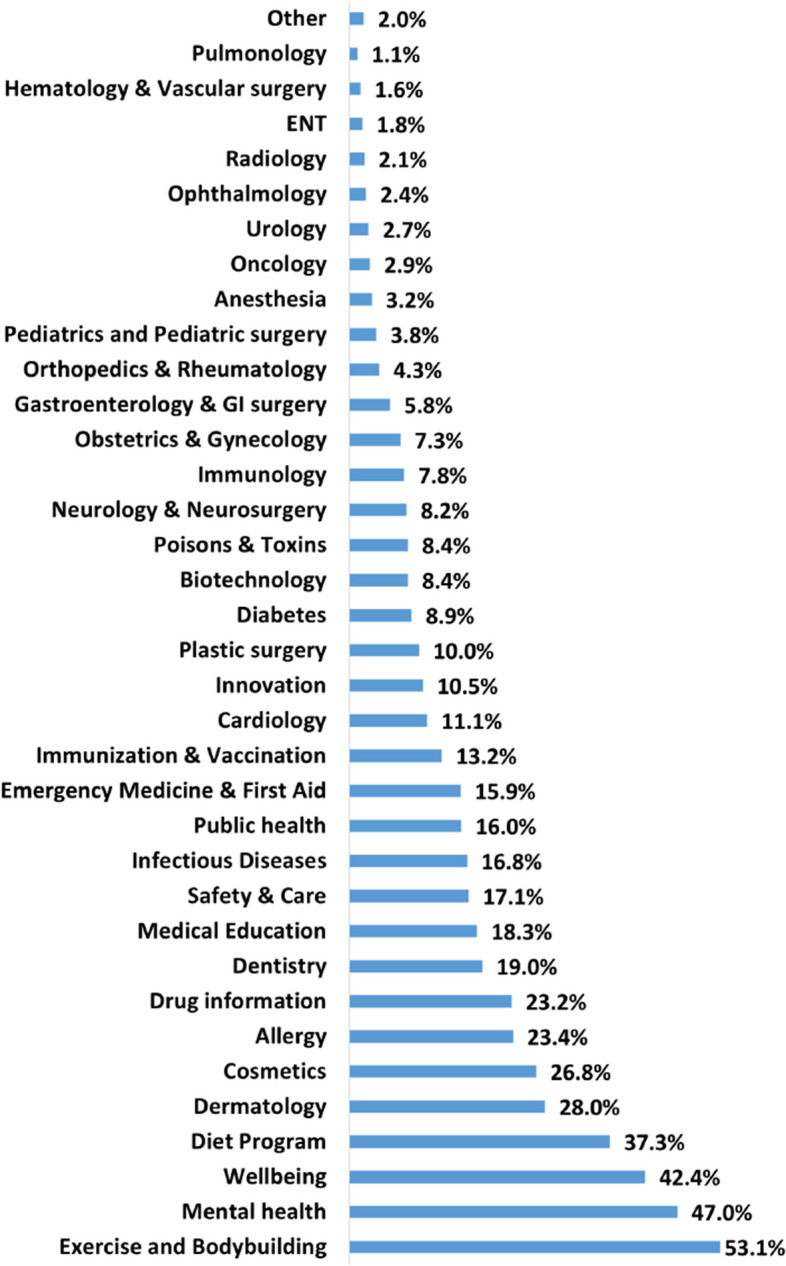
The most watched health-related categories

Figure 4 summarizes the proportions of respondents who watch HRC on YouTube per topic. The diagram shows, for example, that more than half of the participants watch exercise and bodybuilding videos. YouTube is especially attractive for its wide offer of “How to” videos or what is referred to as procedural learning in general [29]. Interestingly, however, when it comes to bodybuilding and fitness videos, research indicates that such content only motivates followers who are already physically active [30]. On the other hand, videos related to Pulmonology and Hematology were among the least viewed ones.

Table 1 summarizes some descriptive statistics related to the number of topics watched by the participants. Accordingly, the average user watches videos related to 5.8 categories with a standard deviation of 3.8 and a median of 5.

**Table 1:** Summary statistic of Watched categories per user

Mean	Median	Mode	SD	Min	Max
5.8	5	4	3.8	1	35

### RQ2-How many users make decisions based on HRC on YouTube, how frequently, and in what fields?

**Fig. 5:**
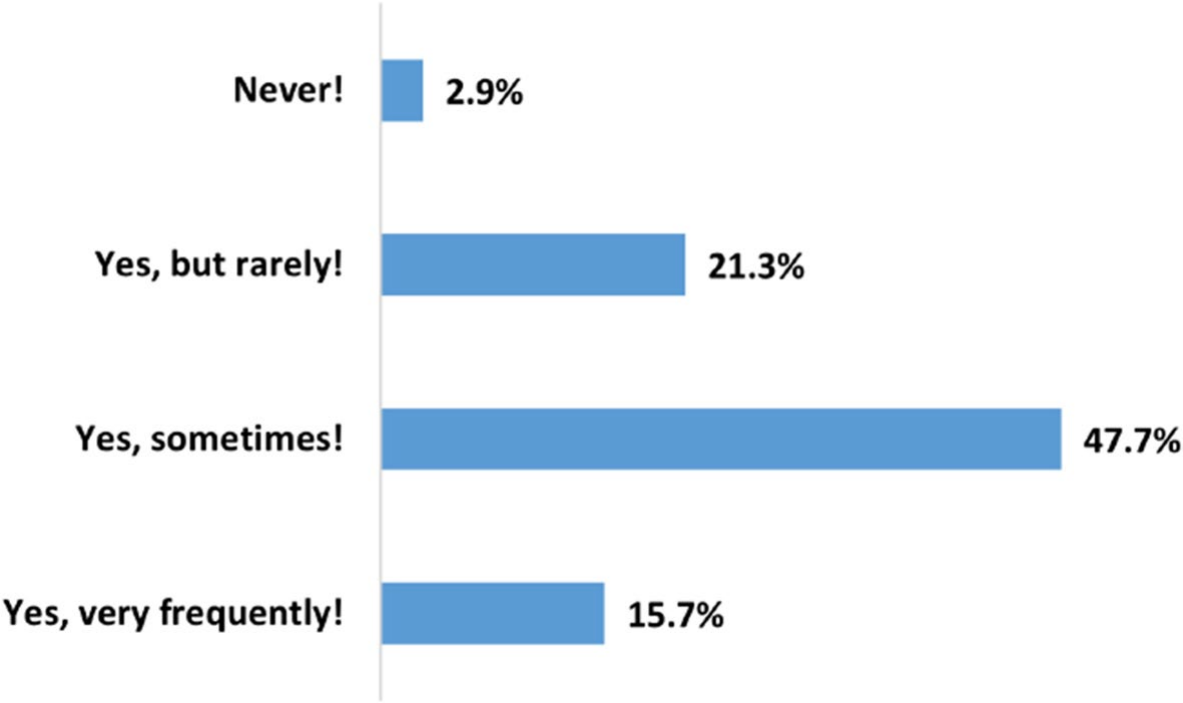
How frequent do users make decisions based on a watched health-related videos on YouTube

When it comes to decision-making, the data in Fig. 5 illustrates that HRC on YouTube impacts 84.7% (n=2542) of the participants. 15.7% (n=470) of the users even make health-related decisions based on YouTube videos very frequently.

**Fig. 6:**
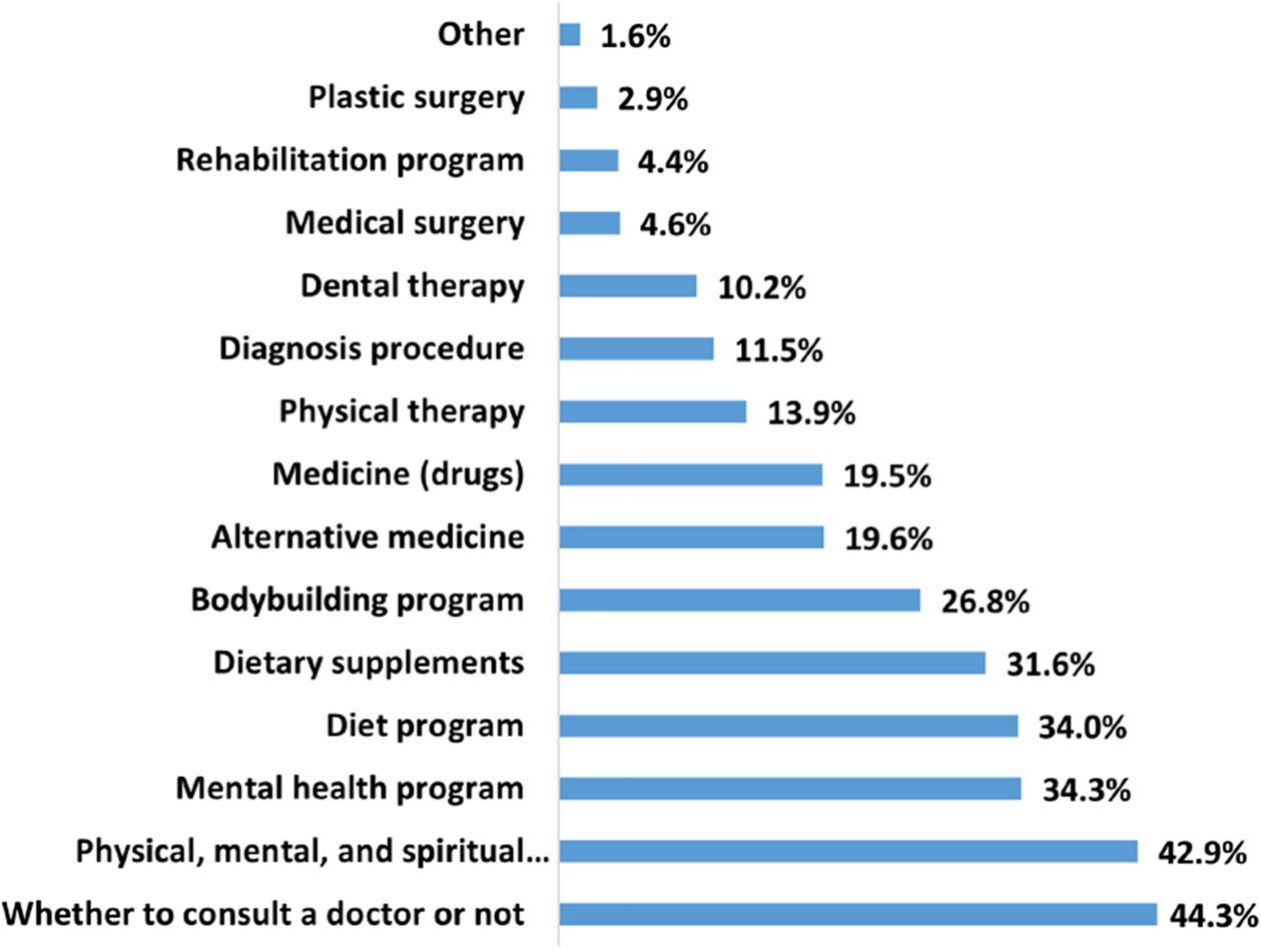
Health-related Decision Categories on YouTube

According to Fig. 6, more than 40% of the users make decisions related to consulting a doctor or adopting certain physical, mental, and spiritual practices (such as yoga, pilates, and gymnastics). Additionally, approximately 34% of the users make decisions related to mental health programs (such as depression and anxiety) as well as diet programs. On average, users make decisions related to 3.6 different categories with a standard deviation of 2, as summarized in Table 2.

**Table 2:** Summary statistic of decision-making categories

Mean	Median	Mode	SD	Min	Max
3.6	3	3	2	1	15

### RQ3-How do users perceive the quality of HRC on YouTube?

**Fig. 7:**
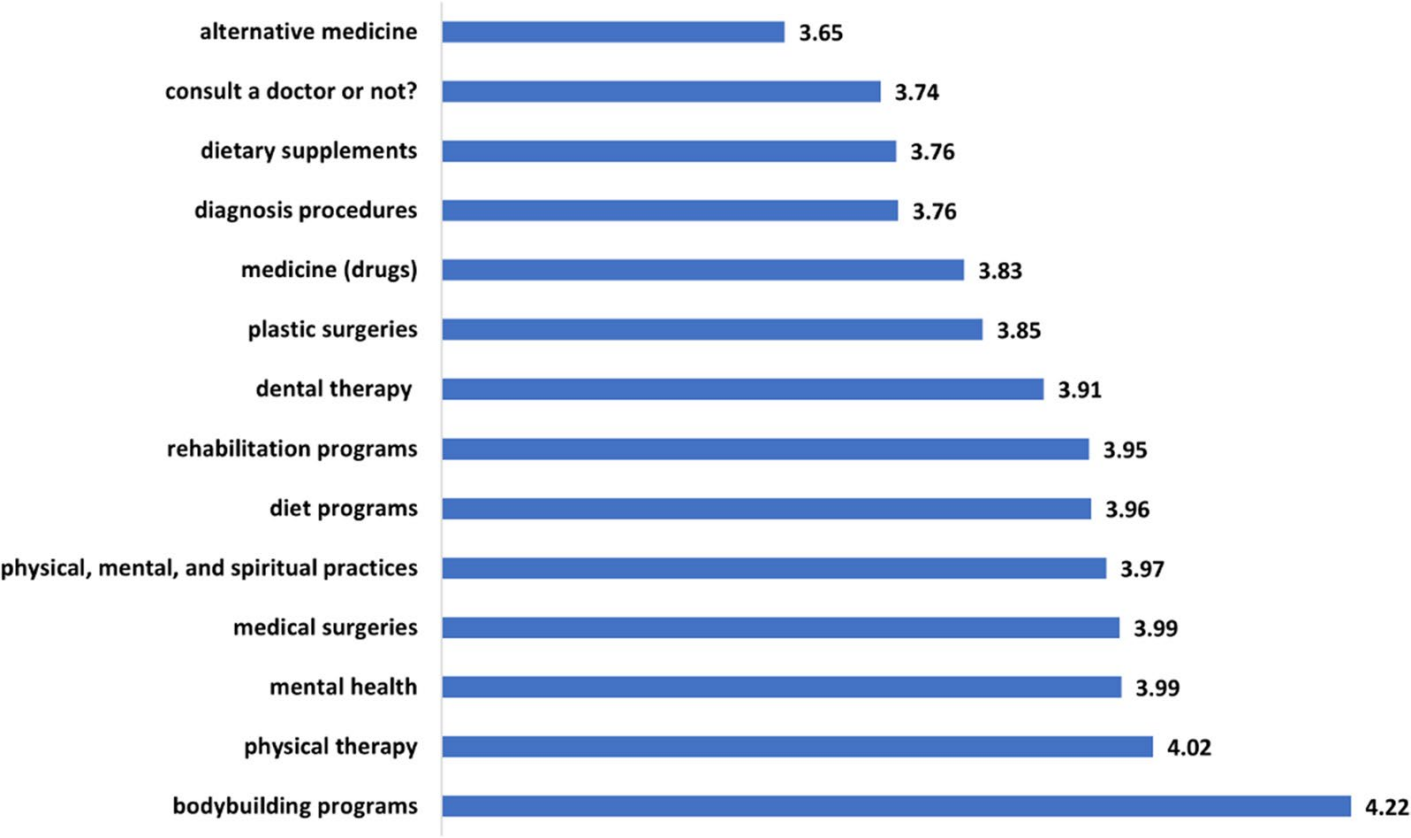
The average rate of the perceived usefulness of the different HRC categories for decision-making

Figure 7 shows the average rate of the perceived usefulness of the videos in the different decision-making categories. Table 3 maps the rating ranges into five levels of perceived usefulness. Accordingly, the users perceive bodybuilding videos as *very useful* for related decision-making, with an average rate of 4.22. The other categories received average rates from 3.65 to 4.02, indicating that they are considered as generally *useful* by the participants.

**Table 3:** Mapping rating ranges to perceived levels of usefulness

Range	Perceived level of usefulness
1.0 - 1.80	Poor or misleading
1.81 - 2.60	Not useful
2.61 - 3.40	Slightly useful
3.41 - 4.20	Useful
4.21 - 5.0	Very useful

**Fig. 8:**
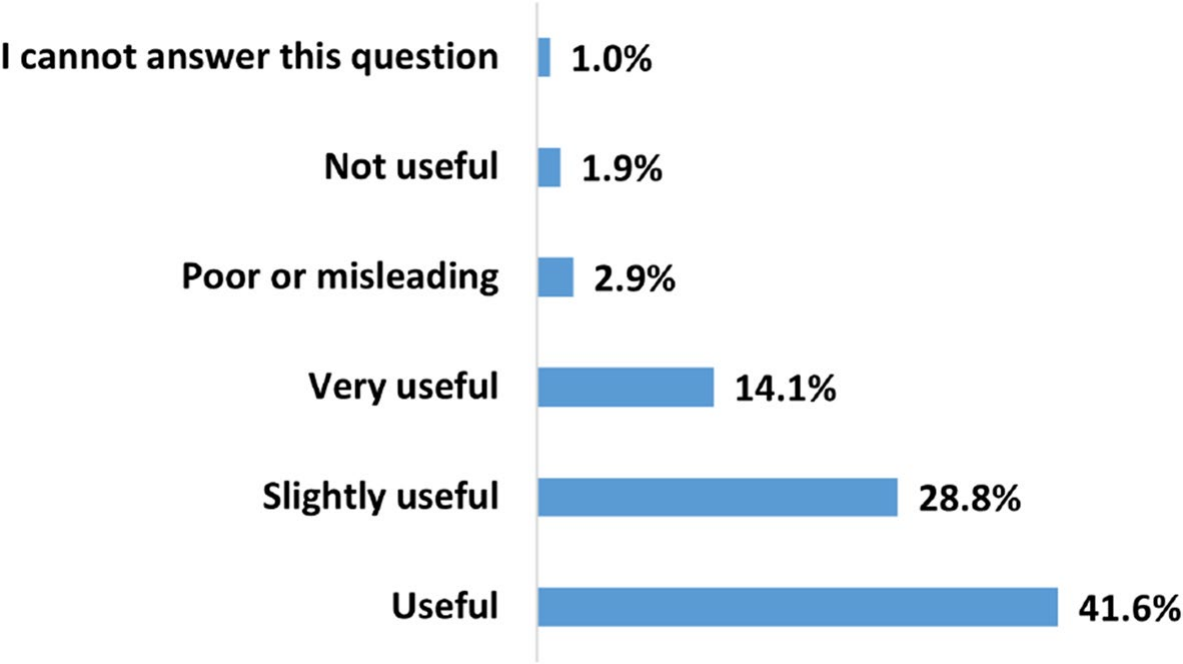
How do users perceive the usefulness of HRC on YouTube

**Fig. 9:**
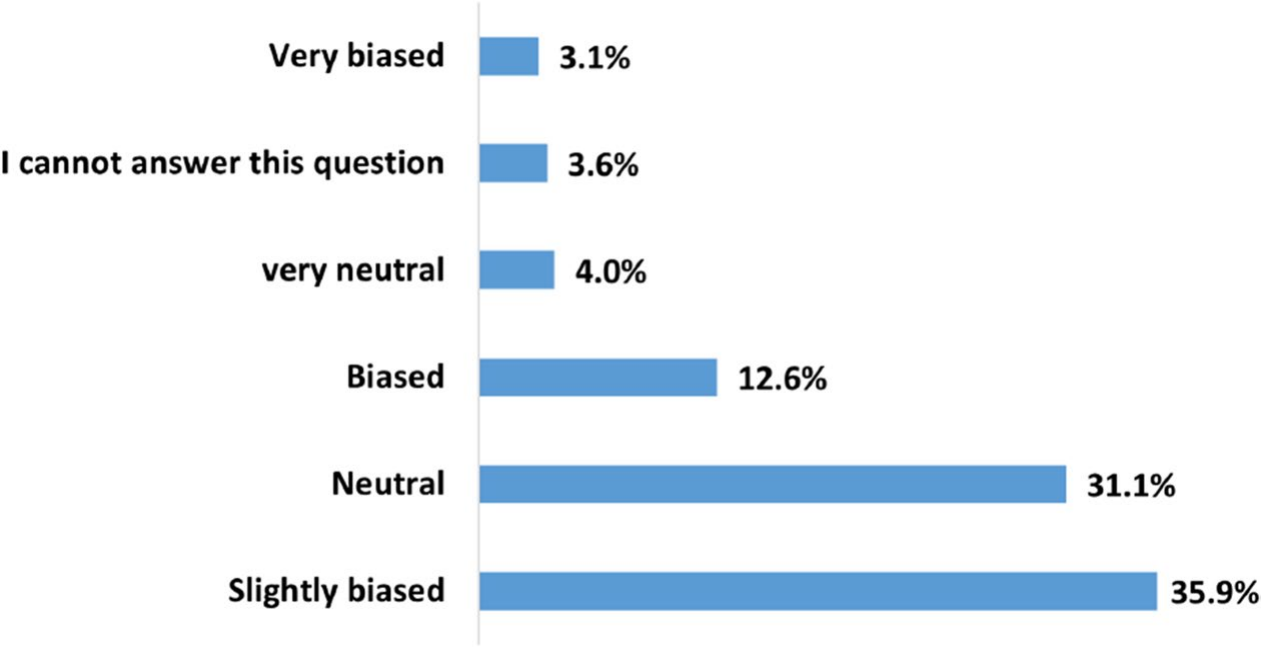
How do users perceive the bias of HRC on YouTube

Figures 8 and 9 summarize the users’ evaluation of the *overall* perceived usefulness and bias of the HRC videos on YouTube. According to Fig. 8, 41.6%, 28.8%, and 14.1% of the survey participants found HRC on YouTube useful, somewhat useful, or very useful, respectively. The overall usefulness score is 3.696, indicating that the majority of respondents perceived the content as useful. This is in line with the overall usefulness scores of the specific decision-making categories mentioned earlier. Regarding bias, Fig. 9 shows that 35.9% of the respondents perceived health-related content on YouTube as somewhat biased, while 31.1% considered it neutral. Only 12.6% of the participants believed that the content is biased. The average overall bias score is 3.233, suggesting that most health-related videos on YouTube are perceived as slightly biased.

### RQ4-How do users link video surface features to content quality?

**Fig. 10:**
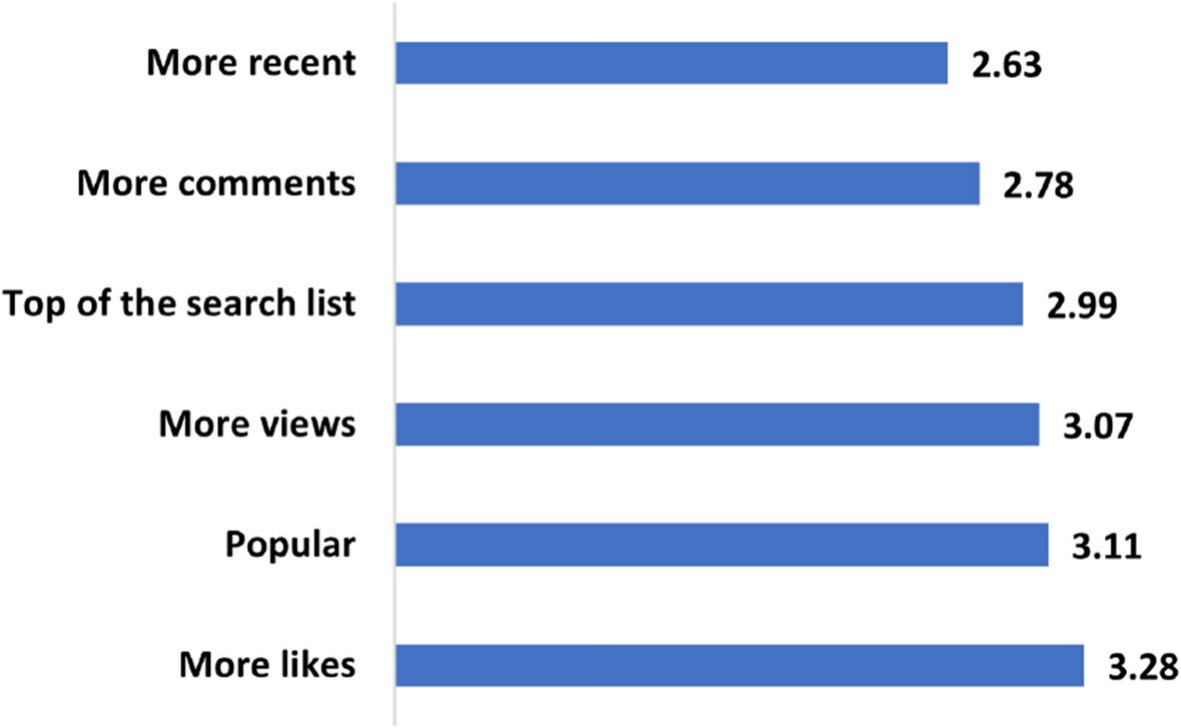
How do the different surface features contribute to content quality from users’ perspective?

Table 4 summarizes users’ responses to the questions (Q25 to Q30) aimed at assessing their perceptions of the links between video surface features and content quality. Accordingly, 32.6% (n=979) of the respondents believe that videos with more likes are of better quality. In contrast, users perceive no link between the number of views and content quality. While 27.6% (n=829) agreed that more views reflect better quality, 25.1% (n=753) held the opposite opinion. On the other hand, most users (35.4%, n=1061) believed that recent videos are not necessarily of higher quality. The respondents remained neutral about the relationship between the video rank or channel popularity and the content quality. This means that the videos that appear at the top of the search list or those from popular channels do not necessarily have better quality. Similarly, 33.4% (n=1003) of users held a neutral view about the link between the number of comments and quality, with 31.0% (n=931) disagreeing that videos with more comments have better quality. The average score of every question is shown in Fig. 10. Table 5 maps these scores to agreement levels. In summary, the findings suggest that video surface features do not impact users’ perceptions of content quality since the averaged scores range from 2.63 to 3.28, i.e., are at the *Neutral* level.

**Table 4:** User perceptions of the relationships between video surface features and content quality

Surface feature	Fully agree	Agree	Neutral	Disagree	Fully disagree
Video rank in search list	4.0%	24.2%	**32.6%**	25.7%	3.8%
Number of views	6.1%	**27.6%**	27.1%	25.1%	4.4%
Number of likes	8.8%	**32.6%**	27.1%	18.4%	3.3%
Number of comments	4.0%	15.7%	**33.4%**	31.0%	6.1%
Video recency	2.7%	14.5%	28.8%	**35.4%**	8.9%
Channel popularity	5.4%	28.3%	**31.6%**	20.6%	4.3%

**Table 5:** Mapping rating ranges to levels of agreement

Range	Level of agreement
1.0 - 1.80	Fully disagree
1.81 - 2.60	Disagree
2.61 - 3.40	Neutral
3.41 - 4.20	Agree
4.21 - 5.0	Fully agree

### RQ5-How does age, gender, profession, or educational level affect the perceived usefulness of HRC on YouTube?

The results of the correlation analysis between the perceived usefulness of HRC and participant characteristics, such as age, gender, profession, and educational level, are presented in Table 6. The Pearson correlation coefficient (*r*) was used to determine the strength of the correlations. The findings indicate that age and profession exhibit weak correlations with the perceived usefulness of HRC. However, the analysis reveals that gender and educational level are not significantly correlated with content quality, as indicated by the large *p*-values.

**Table 6:** Correlation between the perceived usefulness of HRC and the participants age, gender, educational level, and profession

	*r*	*p*-value
Age	-0.12	$<0.001$
Gender	0.03	0.11
Education level	-0.02	0.33
Profession	0.05	0.02

### RQ6-What do users recommend to mitigate the quality issue of HRC on YouTube?

**Fig. 11:**
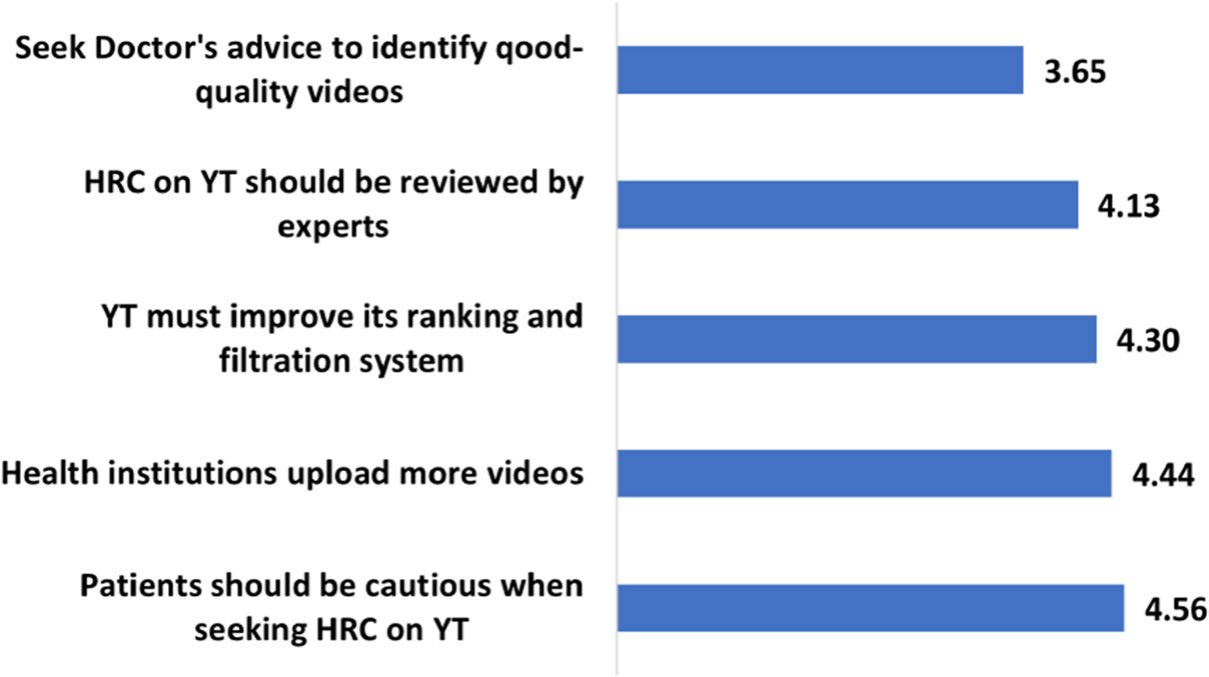
Average likert-scale scores for the recommendations

Table 7 summarizes the users’ agreement or disagreement with various recommendations to enhance the quality of health-related content on YouTube. Accordingly, the most agreed-on recommendation is that users should exercise caution when viewing health videos, with 84.5% (n=2531) of the users either fully agreeing or agreeing. Furthermore, 82.6% (n=2477) of the participants agree or fully agree that reputable sources, such as professional societies and health institutions, should upload more content on YouTube. 78.0% (n=2340) of the users believe that YouTube should enhance its ranking and filtration system to promote high-quality content and drop out misleading videos. 72.8% (n=2184) of them agree or fully agree that health-related content on YouTube needs to be reviewed by experts, and 53.8% (n= 1613) agree or fully agree that patients should seek their doctor’s advice to identify good-quality videos. Figure 11 shows the average score of every recommendation. Accordingly, these scores range from 3.65 to 4.56, indicating that the users either agree or fully agree with the mentioned recommenfations.

**Table 7:** How users perceive the various measures to improve health-related content quality

Recommendation	Fully agree	Agree	Neutral	Disagree	Fully disagree
Patients should be cautious	**56.9%**	27.6%	4.9%	0.9%	0.0%
Health institutions should upload more content	**48.9%**	33.7%	6.5%	1.0%	0.2%
YT should improve its recommending system	**42.3%**	35.7%	10.2%	1.6%	0.4%
Videos should be reviewed by experts	35.7%	**37.1%**	12.5%	3.9%	1.1%
Seek doctor’s advice to identify quality content	19.7%	**34.1%**	24.4%	9.7%	2.4%

## Discussion

Given the widespread use of YouTube as a source of health-related information, it is crucial for researchers and health professionals to comprehend and investigate users’ experiences with HRC videos on the platform. This study aims to contribute to this knowledge by synthesizing current evidence that users are watching HRC videos and making decisions based on what they watch. In addition, it shows how users perceive the usefulness of HRC videos and compares it with published research studies that evaluate the quality of HRC on YouTube.

### RQ1-How many users watch HRC on YouTube, how frequently, and what do they watch exactly?

This study revealed that a significant proportion of users, approximately 87.6%, actively engage with HRC on YouTube, and 16.5% even do this very frequently. This finding aligns with the observed trend of watching YouTube videos for health-related information, as reported in various research studies and national surveys. For instance, the Health Information National Trends Survey (HINTS) documented a notable increase in the percentage of U.S. adults who watched health-related YouTube videos over the years, from 35.3% in 2013 (HINTS 4 CYCLE 3) to 58.9% in 2022 (HINTS 6) [3]. In 2022, 3.3% of U.S. adults reported watching health-related videos on YouTube almost every day, while 7.2% reported watching them at least once a week [3]. Moreover, in a study on testicular pain, Melchionna et al. utilized the Google Trends tool to analyze the growth in YouTube users seeking information on this topic. They observed a substantial increase in the number of users connecting to YouTube for information on testicular pain over the past decade [31].

This study also has shed light on the diverse range of health-related topics that users watch on YouTube. Most of the research papers that were published in the literature looked at how good medical content was in specific fields like Rheumatology and Orthopedics, Gastroenterology and GI surgery, Dentistry, Neurology and Neurosurgery, and Urology [22]. However, people who use the platform are more interested in topics related to exercise and bodybuilding, mental health, well-being, diet programs, and dermatology. It can be noted that the topics considered in previous research tend to be about illnesses and treatments, whereas users are actively looking for health-promoting videos. This might be because the participants in our study were mostly young, around 31.5 years old on average, and many of them went to college. In the future, replication studies should examine this aspect in detail, considering users of older ages and with less academic progress.

### RQ2-How many users make decisions based on HRC on YouTube, how frequently, and in what fields?

Existing literature has highlighted the widespread use of the internet, including platforms like YouTube, for health-related decision-making [32–34]. Our study revealed that a significant majority of users, 84.7%, are making health decisions based on what they watch, with 15.7% doing so even more frequently.

Previous research studies have explored the impact of YouTube health-related videos on users’ decision-making processes, highlighting both positive and negative effects [34–36]. For instance, a study by Ardrini et al. found that watching YouTube videos had a negative influence on college students, leading them to make unhealthy food choices that directly affected their health [34]. Similarly, during the COVID-19 pandemic, Charles E Basch et al. observed a significant increase in videos discussing adverse reactions to the COVID-19 vaccine, which had the potential to influence consumers’ beliefs and decision-making regarding vaccination uptake [37]. On the other hand, several studies have also reported positive impacts on decision-making. For example, Juhan Lee et al. found that watching YouTube videos was associated with a 30% increase in physical activity levels among U.S. adults [4]. This emphasizes that YouTube has the potential to shape the users’ beliefs and decisions [36]. However, some studies caution against using YouTube as a sole basis for decision-making due to potential inaccuracies and misinformation [38]. Thus, numerous studies have emphasized the importance of developing and promoting high-quality content to enhance informed decision-making processes and assist users in making sound choices [36, 39, 40].

The results of this study suggest that most of the users who are watching medical videos on YouTube are making decisions related to whether to consult a doctor or not. In essence, they are matching their symptoms with the information provided in the video to aid their decision-making process. However, this situation carries a potential risk, as some users might decide not to visit a doctor after watching a YouTube video even if their condition truly necessitates professional medical attention. Thus, our study shows that YouTube may influence the health of those who consume health-related videos on YouTube. It is also evident in this study that the decision categories are strongly related to the watching categories as shown in Figs. 4 and 6. This indicates that the participants’ answers were consistent, implying that they are making decisions based on what they watch.

### RQ3-How do users perceive the quality of HRC on YouTube?

This study showed that users perceive the overall quality of HRC videos on YouTube as useful but slightly biased. This does not align with the compiled results of 202 research papers presented by Osman et al., where a majority of health-related videos on YouTube were categorized as not useful or of poor quality according to researchers and experts [22], indicating a disparity between user perceptions and expert evaluations of content quality.

Bias is a complex and multifaceted aspect of medical research, education, and practice, as well as pharmaceutical marketing. The literature addresses cognitive biases linked to medical decisions, associating them with overconfidence, the anchoring effect, information availability, and risk tolerance [41]. Implicit, unconscious bias has also been verified in healthcare provision, leading to suboptimal treatment, inaccurate diagnoses, or delays based on irrelevant factors like race or gender [42]. Furthermore, information bias in health research has been identified and analyzed [43], and bias in pharmaceutical marketing is examined due to the inherent incentives for drug sales representatives to overly endorse their products [44]. Interestingly, cognitive biases in consumer health information seeking have also recently gained attention [45]. Given its complexity, when the participants were asked to rate the bias, they were not provided with any specific contextual details, prompting them to offer a broad, overall assessment. Indeed, it would be very useful to answer the following questions: 1) What specific biases came to the participants’ minds when assigning their ratings? Did their considerations involve cognitive biases, potentially reflecting the presenter’s inclination toward particular diagnostic methods or treatments? Or were they more indicative of marketing biases, possibly geared towards the promotion of specific drugs, etc.? 2) Were the participants themselves influenced by any biases while evaluating bias within HRC? Answering these questions deserves dedicated studies that take the HRC category into consideration.

The disparity in evaluating usefulness and bias was further analyzed by conducting a correlation analysis. The results have revealed a statistically significant, though low, negative correlation between users’ perceptions of usefulness and bias (r = -0.19, p = $p=6\times 10^{-23}$). So, users who rated usefulness higher tended to rate bias lower. This at least mitigates the controversy of the discrepancy in the mean values. Still, more in-depth analysis should be performed in future studies.

It should be highlighted that the study does not aim to assess HRC quality based on users’ judgment. Rather, we aimed to demonstrate that YouTube users, likely due to their lack of expertise, not only watch HRC but also make decisions based on what they watch. According to technology acceptance theories, users must have motivations for using technology [46], with perceived usefulness as the primary motivation. Recalling that 87.6% and 84.7% of our participants watch and make decisions, respectively, and that the overall usefulness score they gave is 3.62/5 (useful), the study indicates that the users’ assessment of HRC usefulness is at least consistent with their watching and decision-making behaviors which aligns with technology acceptance theories.

### RQ4-How do users link video surface features to content quality?

The findings of this study indicate that users’ perceptions of content quality on YouTube are not significantly influenced by videos’ surface features, ranking, or popularity. This aligns with the compiled results of several research studies, which consistently demonstrate that there is no correlation between the number of views, likes, and the overall quality of the videos [6, 22].

This finding suggests that users are aware that popular videos are not always of high quality and they are not solely relying on metrics like view counts or likes when assessing the quality of information. Instead, they may be employing other criteria such as the credibility of the source and the overall usefulness and relevance to their specific health needs. Nevertheless, it is important to acknowledge that low-quality, non-credible videos seem to be more prominent to users compared to trustworthy videos [47, 48]. Thus, professional content creators should consider popularity factors to gain more visibility on the platform.

### RQ5-How does age, gender, profession, or educational level affect the perceived usefulness of HRC on YouTube?

Part of this study aimed to explore the relationship between the socio-demographic characteristics of the participants (age, gender, profession, and educational level) and the perceived usefulness of HRC on YouTube. Our findings revealed weak correlations between age and profession with the perceived usefulness of HRC, while gender and educational level showed no significant correlation. Previous research has explored the influence of various demographic factors on online health information-seeking behavior [49–51]. For instance, studies by Rice and Demirci et al. have highlighted that age, gender and education were significant factors associated with online health information-seeking behavior. Both studies showed that Health information was sought more frequently by women who are young and highly educated [50, 52]. Richmond and Frances found that gender, educational level, and monthly income were significant factors associated with online health information-seeking behavior, while age did not show a significant association [49]. Additionally, Aisha et al. found that younger individuals with a history of heart disease, hypertension, and diabetes, were more likely to watch HRC videos on YouTube compared to older individuals. Furthermore, additional associations related to gender and education were observed among those with hypertension [53]. While these studies focus on identifying the influence of socio-demographic characteristics and the online HRC seeking behaviours of the users, our study try to investigate if the perceived usefulness of the HRC video of YouTube is directly influenced by these characteristics. While our findings reveled a weak or none significant correlations between the socio-demographic characteristics with the perceived usefulness of HRC videos on YouTube, we believe that it is important to consider the intricate relationship between participants’ characteristics, their seeking behaviour and their perceptions.

### RQ6-What do users recommend to mitigate the quality issue of HRC on YouTube?

The findings of this study indicate that users are aware of the existence of low-quality health-related content (HRC) on YouTube. In particular, almost 85% of them recommend being cautious while watching HRC videos on the platform. Paradoxically, however, the participants perceive HRC videos as useful, rating them 3.6 out of 5. While this appears to be inconsistent with their general perception of usefulness, it can be explained by egocentric anchoring bias to an extent: While users’ general assessment of HRC usefulness can be based on what they usually watch, their evaluation of detailed aspects is probably based on experience with content that they would avoid. The following trivial example explains this. A consumer could assess an electronic store as useful because it has some high-quality stuff that the consumer needs and buys, although the store has other products that have not been reviewed and other low-quality items. We did not find literature that describes this behavior exactly but the work by Elpey et al. shows that people adopt others’ perspectives by serially adjusting from their own [54].

Users recommend that YouTube should have more content from reputable sources, such as professional health institutions. This suggests that users value content from trusted and authoritative sources, as they are more likely to provide accurate and reliable health information. While users prioritize caution and reputable sources, previous studies, such as Osman et al. [22], have emphasized the importance of reputable resources and expert guidance in selecting high-quality content. It should be noted that uploading more videos from reliable sources can be less effective if these videos are not promoted by the YouTube algorithm. Finally, users recommend involving experts in reviewing and endorsing HRC videos on YouTube. This would require a significant change in the YouTube system to enable experts’ access, review, and comments, as well as to upgrade the search and ranking system to prioritize expert-endorsed videos.

### Study implications

The following are some take-home messages for YouTube medical content consumers, producers and researchers.

Users should exercise caution when watching HRC videos on YouTube and should not rely solely on YouTube as a source for making health-related decisions. Consulting a medical professional is an essential step towards diagnosis and treatment, and it should be considered prior to making relevant health-related choices.

Credible content creators, such as medical institutions and health agencies, should create and upload more videos on YouTube. However, they need to take popularity factors into account to gain attention on the platform. To achieve this, they should develop interesting and visually appealing videos that capture viewers’ attention. This approach will help enhance the visibility of high-quality HRC videos on YouTube.

This study showed that the users aren’t sure about the relationship between the popularity and quality of HRC on YouTube. However, the study didn’t explore how users behave according to this aspect. In other words, we don’t know how users select and evaluate medical content on YouTube. More research is needed to investigate such behaviors and to study the relationship between popularity and quality [55]. This exploration could potentially lead to the formulation of technical systems employing quality-based algorithms to enhance the visibility of high-quality health-related videos. Furthermore, this study has identified the topics that users find relevant to their health-related decisions. These topics should be considered and prioritized in future research studies.

### Study limitations

Our study had several limitations. Firstly, we only considered English-speaking individuals due to the fact that English is the predominant language used on YouTube [56, 57]. While this decision was based on practical considerations, it may have introduced a language bias in our sample. Secondly, the use of the Prolific platform has introduced another limitation. Based on the platform’s participation strategy, survey spots are allocated on a first-come, first-served basis [58]. This means that the participants who were online and available at the time of survey publication were more likely to participate. However, we believe that the impact of this limitation on our results was mitigated by the large sample size and the fact that we did not impose any restrictions on the participant’s country of residence. Another limitation is that most of our participants are young (with an average age of 31.5 years) and have gone to college. This suggests that the sample is not evenly distributed across different ages and educational levels. In addition, the study would benefit from a refined question about the participants’ educational backgrounds e.g., to include a category for trade or technical diplomas.

In this study, the watching categories were identified based on a previous systematic review [22], as an inclusion criterion. Since this previous review does not address decision-making, new categories were added to the decision-making list based on experts’ consultation and the feedback obtained during the survey testing with 15 participants. Future studies may benefit from including more categories such as nutrients, herbs, and supplements or complementary and alternative medicine.

The watching and decision-making categories were specified using medical terms. The decision to retain certain terms, despite the potential challenge for laypersons, was motivated by two considerations: 1) Some challenges were encountered in identifying alternative terms or crafting concise descriptions that would maintain the precision of the original terminology. 2) Trust was placed in the reliability of Prolific participants, given the platform’s robust quality assurance system. Specifically, it was anticipated that users with relevant experience pertaining to certain terms would be equipped to recognize and interpret them. For instance, individuals with respiratory system issues would likely identify the term “Pulmonology”. In contrast, participants unfamiliar with certain terms might either conduct web searches for clarification or choose to skip them. While this approach is not flawless and represents a limitation in our study, we believe it strikes a balance between the need for precision and the conciseness of the questions.

This research represents a preliminary exploration of user perceptions and decision-making after watching HRC on YouTube. This study didn’t investigate the motivation and methods that users utilize to judge the quality of HRC videos or to make decisions based on them. This work lays future directions for more comprehensive investigations into the motivations behind video consumption, the factors contributing to perceived usefulness, and the varying influences on users’ decision-making based on different categories and demographics. We acknowledge this limitation as an opportunity for future in-depth research in understanding user behaviors and preferences in the context of health-related content on YouTube.

### Future work

This study serves as an exploratory endeavor to investigate how users perceive HRC content on YouTube. Given the evolving nature of this field, as evidenced by the current state of literature, there are promising avenues for future research. Understanding user behaviors and the criteria employed for assessing online content quality, as well as investigating their perceptions of the correlation between popularity and content quality, remains an interesting area for exploration. Another area of interest is to analyze the motivations behind watching HRC videos on YouTube and the factors leading users to make decisions after watching them. Furthermore, it would be interesting to examine whether health-related fields presented through procedural or demonstrational videos gain higher ratings and acceptance among users in comparison to conceptual videos focusing on disease definitions and symptoms.

## Conclusion

YouTube has long been an important source of health-related information. This study extends our understanding of its significance for public health: users turn to YouTube not only to gain knowledge but also to make decisions. Given what we know from the literature about the prevalence of poor and misleading videos and what this study revealed about users’ perceptions of content quality, the findings suggest that YouTube users are at considerable risk of making wrong decisions related to health. This situation is concerning and requires qualitative and quantitative research to explore in detail what motivates people to use YouTube as a decision-making tool, how it helps them develop their decisions, and how they assess it after making relevant decisions.

### Supplementary Information

**Additional file 1.**

## Data Availability

The data for this study is available from the corresponding author upon request via email.

## Abbreviations

APHA: American Public Health Association
CMSS: Council of Medical Specialty Societies
HINTS: Health Information National Trends Survey
HRC: Health-related content
JAMA: Journal of American Medical Association
NAM: National Academy of Medicine
ProA: Prolific Academic
WHO: World Health Organization
