# Supplementary material for: Users’ experience with health-related content on YouTube: an exploratory study
Source: BMC Public Health. 2024 Jan 3;24:86. doi: 10.1186/s12889-023-17585-5 (PMC10765842; doi:10.1186/s12889-023-17585-5)
Supplement: Supplementary file 1 — Additional file 1. [file 12889_2023_17585_MOESM1_ESM.pdf]

Appendix A Survey Questions

A.1 Survey Part 1

1 Please enter your Prolific ID: *Type your answer here*

2 What is your highest educational degree?
  a) Elementary school
  b) High school
  c) Bachelor
  d) Master
  e) Doctorate
  f) Other

3 Is your profession or study field related to health or medicine?
  a) Yes, strongly
  b) Yes, slightly
  c) No

A.2 Survey Part 2

4 Do you watch YouTube videos to seek information related to your health?
  a) Yes, very frequently!
  b) Yes, sometimes!
  c) Yes, but rarely!
  d) Never!

5 In which of the following areas do you watch YouTube videos? *Choose as many as you like*
  a) Allergy
  b) Anesthesia
  c) Biotechnology
  d) Cardiology
  e) Cosmetics
  f) Dentistry
  g) Dermatology
  h) Diabetes
  i) Drug information
  j) Emergency Medicine and First Aid
  k) ENT
  l) Gastroenterology and GI surgery
  m) Hematology and Vascular surgery
  n) Immunization and Vaccination
  o) Immunology
  p) Infectious Diseases
  q) Innovation
  r) Medical Education
  s) Mental health
  t) Neurology and Neurosurgery

- u) Obstetrics and Gynecology
- v) Oncology
- w) Ophthalmology
- x) Orthopedics and Rheumatology
- y) Pediatrics and Pediatric surgery
- z) Plastic surgery
- aa) Poisons and Toxins
- ab) Public health
- ac) Pulmonology
- ad) Radiology
- ae) Safety and Care
- af) Urology
- ag) Wellbeing
- ah) Exercise and Bodybuilding
- ai) Diet Program
- aj) Other

6 Why don't you watch YouTube videos for seeking information related to your health?
- a) Because I prefer to ask a doctor about health-related issues
- b) Because I have concerns about the quality of health-related content on YouTube.
- c) Because I prefer to seek health-related information from professional websites
- d) Other

A.3 Survey Part 3

7 Do you think that watching YouTube videos has supported you in making decisions related to your health?
- a) Yes, very frequently!
- b) Yes, sometimes!
- c) Yes, but rarely!
- d) Never!

8 What were such decisions related to? *Choose as many as you like*
- a) Whether to consult a doctor or not
- b) Diagnosis procedure (e.g., radiology, endoscopy, and lab tests)
- c) Medicine (drugs)
- d) Medical surgery
- e) Dental therapy
- f) Physical therapy
- g) Mental health program (e.g., against depression and anxiety)
- h) Plastic surgery
- i) Rehabilitation program
- j) Diet program
- k) Dietary supplements (e.g., vitamins, minerals, and amino acids)

l) Physical, mental, and spiritual practices (e.g., yoga, pilates, and gymnastics)
m) Bodybuilding program
n) Alternative medicine (e.g., massage, acupuncture, and drinking teas)
o) Other

A.4 Survey Part 4

9 Do you think that YouTube was useful for your decision to consult a doctor or not?
a) Very useful
b) Useful
c) Slightly useful
d) Not useful
e) Poor and misleading
f) I don't know

10 Do you think the information about diagnosis procedures on YouTube was useful?
a) Very useful
b) Useful
c) Slightly useful
d) Not useful
e) Poor and misleading
f) I don't know

11 Do you think the information about medicine (drugs) on YouTube was useful?
a) Very useful
b) Useful
c) Slightly useful
d) Not useful
e) Poor and misleading
f) I don't know

12 Do you think the information about medical surgeries on YouTube was useful?
a) Very useful
b) Useful
c) Slightly useful
d) Not useful
e) Poor and misleading
f) I don't know

13 Do you think the information about the dental therapy on YouTube was useful?
a) Very useful
b) Useful
c) Slightly useful
d) Not useful

e) Poor and misleading
f) I don't know

14 Do you think the information about the physical therapy on YouTube was useful?
a) Very useful
b) Useful
c) Slightly useful
d) Not useful
e) Poor and misleading
f) I don't know

15 Do you think the information about the mental health program on YouTube was useful?
a) Very useful
b) Useful
c) Slightly useful
d) Not useful
e) Poor and misleading
f) I don't know

16 Do you think the information about plastic surgeries on YouTube was useful?
a) Very useful
b) Useful
c) Slightly useful
d) Not useful
e) Poor and misleading
f) I don't know

17 Do you think the information about rehabilitation programs on YouTube was useful?
a) Very useful
b) Useful
c) Slightly useful
d) Not useful
e) Poor and misleading
f) I don't know

18 Do you think the information about diet programs on YouTube was useful?
a) Very useful
b) Useful
c) Slightly useful
d) Not useful
e) Poor and misleading
f) I don't know

19 Do you think the information about dietary supplements on YouTube was useful?
a) Very useful

b) Useful
c) Slightly useful
d) Not useful
e) Poor and misleading
f) I don't know

20 Do you think the information about physical, mental, and spiritual practices on YouTube was useful?
a) Very useful
b) Useful
c) Slightly useful
d) Not useful
e) Poor and misleading
f) I don't know

21 Do you think the information about bodybuilding programs on YouTube was useful?
a) Very useful
b) Useful
c) Slightly useful
d) Not useful
e) Poor and misleading
f) I don't know

22 Do you think the information about alternative medicine on YouTube was useful?
a) Very useful
b) Useful
c) Slightly useful
d) Not useful
e) Poor and misleading
f) I don't know

23 I feel that health-related videos on YouTube are:
a) Very useful
b) Useful
c) Slightly useful
d) Not useful
e) Poor or misleading
f) I cannot answer this question

24 I feel that health-related videos on YouTube are:
a) Very biased
b) Biased
c) Slightly biased
d) Neutral
e) Very neutral
f) I cannot answer this question

A.5 Survey Part 5

In the following questions, you will read multiple statements related to health-related videos on YouTube. Please, tell us how far you agree with each statement!

25 Videos that appear at the top of the search list are of higher quality.
   a) I fully agree
   b) I agree
   c) I am neutral
   d) I disagree
   e) I fully disagree

26 Videos that have more views are of higher quality!
   a) I fully agree
   b) I agree
   c) I am neutral
   d) I disagree
   e) I fully disagree

27 Videos that have more likes are of higher quality!
   a) I fully agree
   b) I agree
   c) I am neutral
   d) I disagree
   e) I fully disagree

28 Videos that have more comments are of higher quality!
   a) I fully agree
   b) I agree
   c) I am neutral
   d) I disagree
   e) I fully disagree

29 Videos that are more recent are of higher quality!
   a) I fully agree
   b) I agree
   c) I am neutral
   d) I disagree
   e) I fully disagree

30 Videos from popular channels are of higher quality!
   a) I fully agree
   b) I agree
   c) I am neutral
   d) I disagree
   e) I fully disagree

A.6 Survey Part 6

31 Health-related content on YouTube should be reviewed by experts.
   a) I fully agree

b) I agree
c) I am neutral
d) I disagree
e) I fully disagree

32 Professional health institutions should upload more videos on YouTube.
a) I fully agree
b) I agree
c) I am neutral
d) I disagree
e) I fully disagree

33 YouTube should improve its ranking and filtration system to promote high-quality content.
a) I fully agree
b) I agree
c) I am neutral
d) I disagree
e) I fully disagree

34 Patients should seek their doctor's advice to identify qood-quality videos.
a) I fully agree
b) I agree
c) I am neutral
d) I disagree
e) I fully disagree

35 Patients should be cautious when seeking health-related information on YouTube.
a) I fully agree
b) I agree
c) I am neutral
d) I disagree
e) I fully disagree
